# Development and Validation of a Mechanistic Model That Predicts Infection by *Diaporthe ampelina*, the Causal Agent of Phomopsis Cane and Leaf Spot of Grapevines

**DOI:** 10.3389/fpls.2022.872333

**Published:** 2022-04-07

**Authors:** Elisa Gonzalez-Dominguez, Tito Caffi, Aurora Paolini, Laura Mugnai, Nedeljko Latinović, Jelena Latinović, Luca Languasco, Vittorio Rossi

**Affiliations:** ^1^Horta s.r.l., Piacenza, Italy; ^2^Department of Sustainable Crop Production (DI.PRO.VES.), Università Cattolica del Sacro Cuore, Piacenza, Italy; ^3^Department of Agricultural, Food, Environmental and Forestry Science and Technology (DAGRI), Plant Pathology and Entomology Section, University of Florence, Firenze, Italy; ^4^Biotechnical Faculty, University of Montenegro, Podgorica, Montenegro

**Keywords:** excoriose, *Vitis vinifera*, alpha conidia, systems analyses, process-based model

## Abstract

Phomopsis cane and leaf spot (PCLS), known in Europe as “excoriose,” is an important fungal disease of grapevines caused by *Diaporthe* spp., and most often by *Diaporthe ampelina* (synonym *Phomopsis viticola*). PCLS is re-emerging worldwide, likely due to climate change, changes in the management of downy mildew from calendar- to risk-based criteria that eliminate early-season (unnecessary) sprays, and the progressive reduction in the application of broad-spectrum fungicides. In this study, a mechanistic model for *D. ampelina* infection was developed based on published information. The model accounts for the following processes: (i) overwintering and maturation of pycnidia on affected canes; (ii) dispersal of alpha conidia to shoots and leaves; (iii) infection; and (iv) onset of disease symptoms. The model uses weather and host phenology to predict infection periods and disease progress during the season. Model output was validated against 11 independent PCLS epidemics that occurred in Italy (4 vineyards in 2019 and 2020) and Montenegro (3 vineyards in 2020). The model accurately predicted PCLS disease progress, with a concordance correlation coefficient (CCC) = 0.925 between observed and predicted data. A ROC analysis (AUROC>0.7) confirmed the ability of the model to predict the infection periods leading to an increase in PCLS severity in the field, indicating that growers could use the model to perform risk-based fungicide applications.

## Introduction

Phomopsis cane and leaf spot (PCLS; known in Europe as “excoriose”) is an important fungal disease that is widely distributed throughout the viticultural regions of Europe (Phillips, 2000; Guarnaccia et al., 2018), United States (Schilder et al., 2005; Nita et al., 2008; Urbez-Torres et al., 2013; Wilcox et al., 2015), South Africa (Mostert, 2000), and Australia (Merrin et al., 1995). PCLS mainly affects shoots and leaves, although all green parts of the grapevines can be infected. On shoots, PCLS forms small, black cortical necrotic tissue mostly in the three to four basal internodes; these necrotic tissues develop into elliptical lesions that may grow together to form irregular, black, crusty, longitudinal cankers; when shoots lignify during the dormant season, infected canes become grayish; and numerous black pycnidia emerge from their surfaces. On leaves, the disease consists of small light-green lesions with irregular margins that develop early in the season, which later on turn dark with surrounding yellow halos. On rachises, symptoms are similar to those on shoots, and berries turn brown and shrivel during ripening (Erincik et al., 2002; Nita et al., 2008; Wilcox et al., 2015). Damage to grapevines includes weakened canes (which may break and be susceptible to frost damage), reduced photosynthesis of leaves, poor fruit development, and premature fruit drop because of rachis infection and fruit rot. Fruit and rachis infections are sporadic and occur only under very high disease pressure but if present can be responsible for important losses (Erincik et al., 2002; Nita et al., 2008; Wilcox et al., 2015).

PCLS was traditionally known to be caused by *Phomopsis viticola. Phomopsis viticola* has also been described as a wood-canker pathogen, causal agent of dead-arm symptoms (Baumgartner et al., 2013; Urbez-Torres et al., 2013). Species of the genus *Phomopsis* were considered to be host specific, and more than 1,000 species have been described based on the assumption of host specificity; this assumption is no longer considered valid (Gomes et al., 2013; Urbez-Torres et al., 2013). In the last decade, the genus has been redefined based on a combination of morphological, cultural, phytopathological, and mating type data, and the teleomorph stage *Diaporthe* became the only name broadly accepted and adopted (Udayanga et al., 2011; Gomes et al., 2013; Guarnaccia et al., 2018). As a consequence of this revision, multiple species of *Diaporthe* have been associated with PCLS and trunk diseases on *Vitis vinifera*, including *D. ampelina*, *D. amygdali*, *D. eres*, *D. ambigua*, and *D. foeniculina*. Among these species, *D. ampelina* is the most prevalent (Baumgartner et al., 2013; Urbez-Torres et al., 2013; Lawrence et al., 2015; Guarnaccia et al., 2018; González-Domínguez et al., 2021).

*Diaporthe ampelina* overwinters in lesions on dormant canes that had been infected during the previous growing seasons (Anco et al., 2012; González-Domínguez et al., 2021). In spring, mature flask-shaped pycnidia produce two types of conidia, i.e., alpha and beta conidia, that are different in shape (Sergeeva et al., 2003; Gomes et al., 2013). The primary inoculum of PCLS consists of the alpha conidia produced by *D. ampelina* pycnidia from budbreak until shortly after the end of bloom (Anco et al., 2012; González-Domínguez et al., 2021); these conidia are responsible for infection of leaves and shoots during cool, wet weather (Erincik et al., 2003; Anco et al., 2013). The function of beta conidia in the disease epidemiology is unclear (Pezet, 1974; Sergeeva et al., 2003; González-Domínguez et al., 2021). The disease is known to be monocyclic, because several primary infections occur through the season but no secondary infections contribute to disease progress (Anco et al., 2012; Wilcox et al., 2015; González-Domínguez et al., 2021). PCLS usually increases in a vineyard over a number of years, leading to a general decline in vine vigor and yield (Hewit and Pearson, 1988).

PCLS is re-emerging worldwide (Phillips, 2000; Wilcox et al., 2015; Caffi et al., 2020), because of three probable factors: climate change, which has advanced seasonal grapevine growth, including the time of budbreak (Caffarra and Eccel, 2011; Molitor and Junk, 2019); changes in the management of downy mildew from calendar- to risk-based criteria that eliminate early-season, unnecessary sprays (Rossi et al., 2019); and the progressive reduction in the application of broad-spectrum fungicides, which although highly effective (Nita et al., 2006a), may negatively affect human health and the environment (Wightwick et al., 2010; Epstein, 2014; Mostafalou and Abdollahi, 2017). At present, PCLS control is still mainly based on repeated treatments with protective broad-spectrum fungicides (mancozeb, dithianon, captan, or metiram), which are more effective than QoI or DMI fungicides (Hewit and Pearson, 1988; Nita et al., 2006a), starting from budbreak or at 2.5 cm shoot growth (Pscheidt and Pearson, 1991; Rawnsley, 2012; Wilcox et al., 2015).

To improve the timing of fungicide applications for control of PCLS, Nita et al. (2006b) developed a simple warning system based on the effect of weather conditions on infection by alpha conidia and on the assumption that inoculum is always present in sufficient amount to initiate the disease (Erincik et al., 2003). However, the production and dispersal of *D. ampelina* alpha conidia have a within-season temporal pattern (Anco et al., 2012; González-Domínguez et al., 2021) that may also affect infection occurrence and severity. The latter finding suggests that the timing of fungicide application should be based not only on weather conditions but also on the production and dispersal of *D. ampelina* alpha conidia.

In the current study, we developed a model for predicting the risk of shoot and leaf infection by alpha conidia of *D. ampelina*. The model was developed with a mechanistic approach, which accounted for the processes resulting in the availability of *D. ampelina* conidia during the season and in repeated infections and disease progress over the season. We evaluated the model in four Italian vineyards during two growing seasons (2019 and 2020), and in three Montenegrin vineyards during one growing season (2020).

## Model Development

### The Biological Processes Addressed by the Model

*Diaporthe ampelina* overwinters as pycnidia that are produced on canes and rachises and that remain viable for more than one season (Anco et al., 2012; González-Domínguez et al., 2021). In spring, pycnidia mature, emerge through the periderm of canes, and exude conidia in a gelatinous mass (the cirrus; Nita et al., 2006b). Alpha conidia in pycnidia are produced at temperatures ranging from 4 to 36°C, with an optimum at 21°C, and their production increases with increasing wetness duration (Anco et al., 2013). Dispersal of alpha conidia is mainly associated with rain events, from budbreak to shortly after the end of bloom (Anco et al., 2012; Wilcox et al., 2015; González-Domínguez et al., 2021). No secondary inoculum is produced during the season (Anco et al., 2012; Wilcox et al., 2015; González-Domínguez et al., 2021).

Alpha conidia can germinate at temperatures ranging from 8 to 30°C (temperatures out of this range have not been evaluated); at 20 to 30°C, 100% of the conidia germinate within 28 h (Sergeeva et al., 2003). Infection of grape leaves and shoots occurs at 5 to 35°C; at optimum temperatures (16 to 20°C), infection occurs with a minimum of 5 h of wetness; and wet periods longer than 10 h are necessary at higher and lower temperatures (Erincik et al., 2003). Rachis, berries, and leaves are susceptible through the growing season, from pre-bloom (GS = 15 of the scale of Lorenz et al., 1995) to veraison (GS = 81; Erincik et al., 2001). Symptoms first appear on leaves and shoots at 7 to 11 days after infection, although the effect of the environment and growth stage on the incubation period is not well understood (Erincik et al., 2001, 2003).

### Model Description

**Figure 1:**
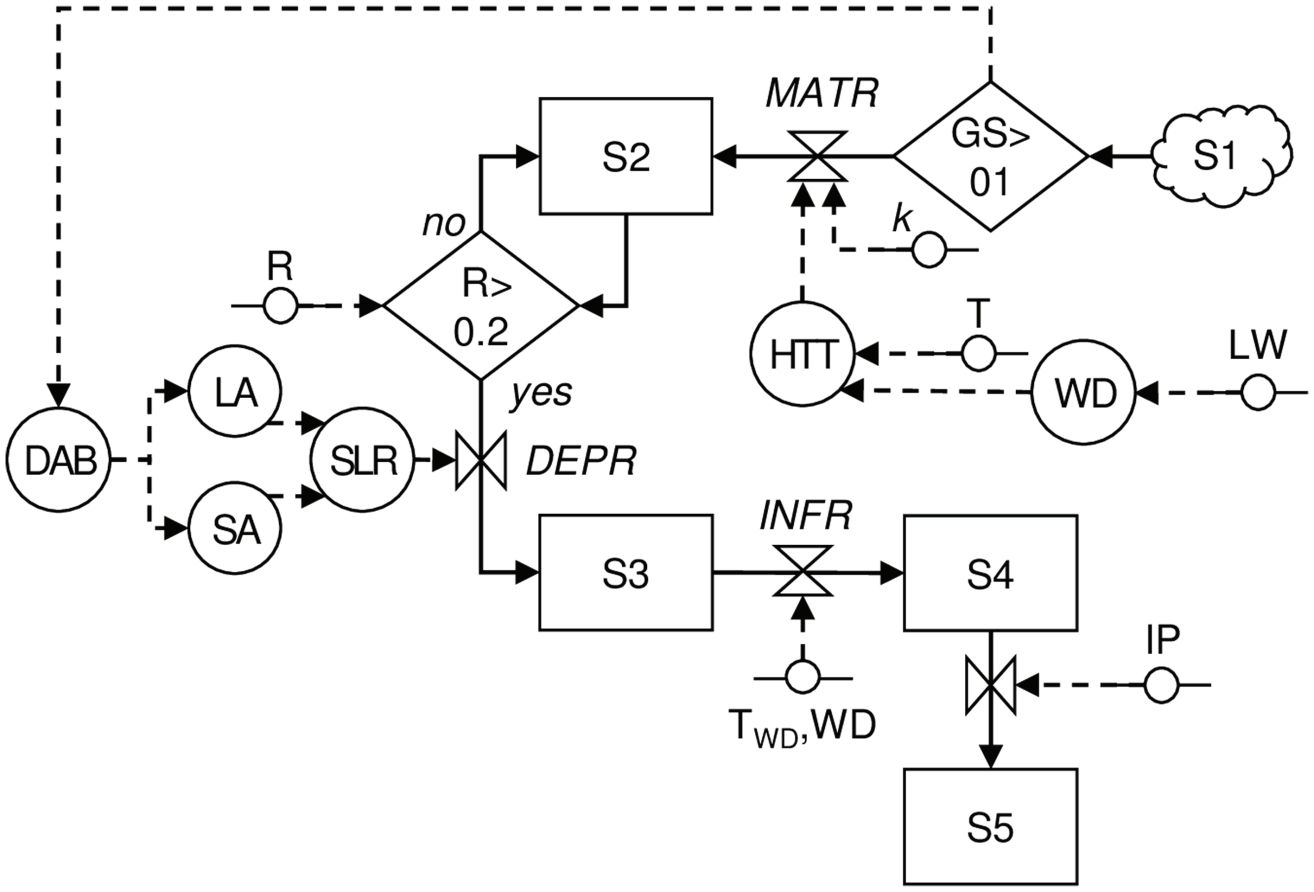
Relational diagram of a model simulating Phomopsis cane and leaf spot (PCLS) dynamics. Boxes are state variables; line arrows show fluxes and direction of changes from one state variable to the next one; valves define rates regulating these fluxes; diamonds show switches (i.e., conditions that open or close a flux); segments with circles indicate external variables; circles indicate auxiliary variables; broken arrows link external or auxiliary variables to diamonds or circles that they influence; and clouds indicate state variables that enter or exit the system (and are not quantified). All variables are listed in Table 1.

The model was developed according to the principles of “systems analysis” (Leffelaar, 1993; Rossi et al., 2010). The biological processes described above were organized into different state variables, and changes from one state to another were determined by rate variables depending on host growth stage and environmental conditions, i.e., temperature (T, °C), rainfall (R, mm), and leaf wetness (LW, min), as shown in a relational diagram (Figure 1; Table 1). The model calculations begin at budbreak (GS = 01) and end at harvest (GS = 90), and the time step is 1 day.

**Table 1:** List of variables, rates, and parameters used in the model.

Abbreviation	Description	Unit
*State variables*		
S1	Overwintering population of pycnidia	N
S2	Population of mature alpha pycnidia	N
S3	Dose of alpha conidia dispersed to the plant	N
S4	Proportion of alpha conidia that cause infection	N
S5	Proportion of alpha conidia that produce symptomatic infections	N
*Rate variables*		
MATR	Maturation rate of conidia	N
DEPR	Deposition rate	N
INFR	Infection rate	0 to 1
*Parameters and auxiliary variables*		
*k*	Modulator of the inoculum level in the vineyard	N
HTT	Hydrothermal time	°C/day
SLR	Shoot-to-leaf ratio	N
LA	Leaf area	cm^2^
SA	Shoot area	cm^2^
DAB	Days after budbreak	Days
IP	Incubation period	Days
*Driving variables*		
T_WD_	Average temperature of the wetness period	°C
GS	Growth stage of vines based on Lorenz et al. (1995)	0 to 99
WD	Duration of the wet period	Hours
T	Temperature	°C
LW	Leaf wetness	0 to 1
R	Hourly rainfall	mm/h

The first state variable in Figure 1 consists of a population of pycnidia (S1, which is not defined in size) that overwinters in the vineyard and produces mature alpha conidia (S2, which is the second state variable in Figure 1). The mature alpha conidia are produced during the grape-growing season starting at budbreak and at a maturation rate (*MATR*).

The maturation rate of conidia is calculated as the first derivative of the equation of González-Domínguez et al. (2021):


(1) $$\mathrm{MAT}R_{i}=k\;e^{-5\;e^{-0.297\;{HTT}_{i}}}$$

in which *k* is a modulator of the inoculum level in the vineyard, and HTT_i_ is the hydrothermal time accumulated on each ith day after budbreak (Lovell et al., 2004). HTT is calculated as follows:


(2). $${HTT}_{i}=\overset{n}{\underset{i=1}{\sum }}79.93\;{{Teq}_{i}}^{3.89}{\left(1-{Teq}_{i}\right)}^{2.83};\;\mathrm{if}\;{WD}_{i}\leq 6\;\mathrm{then}\;{Teq}_{i}=0$$

in which Teq_i_ is an equivalent of temperature calculated as Teq_i_ = (T_i_ – Tmin)/(Tmax – Tmin), where T_i_ the average temperature of the day i, and Tmin and Tmax are the minimal and maximal temperatures for the production of conidia (5 and 35°C, respectively; González-Domínguez et al., 2021); WD_i_ is the wetness duration (i.e., the number of wet hours in a day).

Alpha conidia in S2 are dispersed when rain is ≥0.2 mm/h and a minimum of 24 h have passed since the previous rain (Nita et al., 2006b; González-Domínguez et al., 2021); in the model, this is considered a dispersal event. The model assumes that the dose of conidia that is deposited on shoots or leaves depends on the shoot and leaf area (the auxiliary variables SA and LA, respectively), and a deposition rate (DEPR) that in turn depends on the shoot-to-leaf ratio (the auxiliary variable SLR, calculated as SA/(LA + SA)). Both SA and LA depend on the number of days after budbreak (DAB; Supplementary Material).

The conidia in S3 cause infection when there is a wet period of at least 5 h with a temperature from 5 to 35°C (Erincik et al., 2003). This is an infection event in the model; two infection events are separated by a dry period that is >4 h (Nita et al., 2006b). The severity of any infection event, i.e., the proportion of S3 that causes infection (S4, the fourth state variables in Figure 1), is calculated based on an infection rate for leaves (*INFR_L_*) and shoots (*INFR_S_)* as follows:


(3) $$INFR_{L}=11.23\;{{Teq}_{wd}}^{1.55}{\left(1-{Teq}_{wd}\right)}^{1.84}e^{-9.34\;e^{-0.26\;WD}}$$

(4) INFRS=1.85eTwd−150.591.44/1+eTwd−151.44WDWDmax

in which *T*_wd_ is the average temperature of the wetness period; Teq_wd_ is as in equation (2), with *T* = *T*_wd_; WD is the consecutive number of hours of wetness; and WDmax is the number of wet hours for maximum disease development on shoots (i.e., 20 h). Equations (3) and (4) were developed as described in Supplementary Material.

S4 flows to S5 (the fifth state variable in Figure 1) after an incubation period of 12 days (range 7–18 days) for leaves and 18 days (range 11–25 days) for shoots. Because clear information has not been published (Erincik et al., 2002; Nita et al., 2006b), we defined the incubation period empirically, as shown in Supplementary Material.

### Model Output

Starting from budbreak of the vines (GS = 01), the model produces the following daily outputs: the proportion of the seasonal dose of conidia that (i) is not yet mature (and will mature later in the season), i.e., *k*-S2; (ii) is dispersed to plants at each dispersal event, i.e., S3; and (iii) causes infection on leaves and shoots at each infection event, i.e., S4. The accumulation of S5 over the season provides an estimate of disease progress.

## Model Validation

### Vineyards and Disease Assessment

Disease assessments for model validation were carried out in four Italian vineyards during two growing seasons (2019 and 2020) and in three Montenegrin vineyards during one growing season (2020), for a total of 11 epidemics (Table 2).

**Table 2:** Summary of the characteristics of the vineyards used to validate the *Diaporthe ampelina* model.

Code of epidemic	Vineyard locality (country)^a^	Coordinates	Cultivar	Training system	Establishment year	Period of assessment	N. leaves/shoots evaluated^b^	Budbreak^c^	Final incidence/severity^d^		*k* parameter^e^
									Leaves	Shoots	
DO-2019	Donnini (IT)	43°44′26.9″N 11°30′01.4″E	Sangiovese	Spur cordon	1988	04/04/2019 02/07/2019	669/669	01/04	0.08/0.5	0.06/0.4	0.90
DO-2020						14/05/2020 04/06/2020	821/844	18/04	0.17/2.6	0.08/1.6	2.25
BS-2019	Borgo S. Lorenzo (IT)	43°56′49.8″N 11°20′30.3″E	Sangiovese	Spur cordon	2001	04/04/2019 02/07/2019	1125/1125	01/04	0.04/0.2	0.11/0.9	1.25
BS-2020						07/05/2020 04/06/2020	840/821	13/04	0.08/1.3	0.09/1.6	0.75
PI-2019	Piacenza (IT)	45°02′16.0″N 9°43′40.1″E	Barbera	Double guyot	2011	06/05/2019 26/06/2019	358/380	01/04	0.36/2.3	0.47/6.4	1.10
PI-2020						07/05/2020 20/07/2020	119/231	07/04	0.26/1.3	0.41/2.6	1.10
SO-2019	Sorbara (IT)	44°45′26.1″N 11°00′45.1″E	Lambrusco	Sylvoz	2004	23/05/2019 28/06/2019	149/380	09/04	0.11/0.5	0.23/1.2	0.45
S0-2020						09/05/2020 18/07/2020	279/218	20/04	0.45/2.3	0.55/3.0	2.50
BP-2020	Balabansko Polje (MN)	42°19′13″ N 19°14′43″ E	Vranac	Single Guyot	2008	18/04/2020 15/07/2020	843/843	31/03	0.35/20.5	0.36/7.1	1.60
GO-2020	Godinje (MN)	42°26′46″ N 19°12′21″ E	Vranac	Double cordon	1988	18/04/2020 16/07/2020	1009/1009	29/03	0.24/8.4	0.20/1.9	1.00
PO-2020	Podgorica (MN)	42°26′46″ N 19°12′21″ E	Vranac	Double Guyot	2003	13/04/2020 09/07/2020	1730/1730	29/03	0.26/6.7	0.31/3.4	2.20

a Country: IT, Italy; MN, Montenegro.

b In each epidemic, the internodes and leaves of 9–13 plants were assessed (8–10 shoots per plant).

c Day and month when budbreak occurs (GS 01 of the scale of Lorenz et al., 1995).

d Incidence rated on a 0–1 scale as the average of the proportion of internodes or leaves with PCLS symptoms. Severity on leaves rated on a 0–100 scale as follows: 0 = healthy leaf; 5 = only a few, isolated spots on the leaf; 15 = many spots on the leaf, but still isolated; 40 = many lesions, and the leaf begins to deform; 70 = many lesions, and the leaf is moderately deformed; and 100 = many lesions, and the leaf is highly deformed. The assessment scale for shoots was as follows: 0 = healthy internodes; 5 = only a few, small, and isolated lesions; 15 = few lesions affecting 10–30% of the surface; 40 = many lesions affecting 30–50% of the surface; 70 = many lesions affecting 50–75% of the surface; and 100 = many lesions affecting the entire internode.

e Constant parameter *k* of equation (1), which accounts for the quantity of *D. ampelina* alpha conidia that can develop from overwintering pycnidia. The parameter was estimated as indicated in section “Data Analysis.”

All of the vineyards had a history of PCLS symptoms and positive detection of *D. ampelina* (Guarnaccia et al., 2018; González-Domínguez et al., 2021). The vineyards were managed following standard practices, and no treatments for control of PCLS were applied. The weather stations were located at 2 m above the ground in the vineyards and provided hourly records of air temperature (T, °C), relative humidity (RH, %), rainfall (R, mm), and leaf wetness duration (WD, min). iMeteos stations (Pessl Instruments GmbH, Weiz, Austria) were used in Italy, and Vantage Pro2 stations (Davis Instruments, Hayward, CA, United States) were used in Montenegro.

Starting from budbreak, the vineyards were inspected to determine (i) the prevalent growth stage of the vines based on the scale of Lorenz et al. (1995) and (ii) the incidence and severity of PCLS on leaves and shoots. In 2019, the assessments were performed until the end of June (80–90 days after budbreak, DAB); based on the observations of 2019, the assessments were extended until late July (100–110 DAB) in 2020 (Table 2). Each year, 8 to 10 shoots were tagged on 9 to 13 plants per vineyard, and all internodes and leaves were observed at 7- to 14-day intervals; in total, 2,554 and 2,114 internodes, and 2,059 and 2,301 leaves were assessed in 2019 and 2020, respectively (Table 2). Disease severity on leaves was rated on a 0–100 scale with interval continuity as follows: 0 = disease-free leaf; 5 = only a few, isolated spots on the leaf; 15 = many spots on the leaf, but still isolated; 40 = many lesions, and the leaf has begun to deform; 70 = many lesions, and the leaf is moderately deformed; and 100 = many lesions, and the leaf is heavily deformed. Similarly, disease severity on shoot internodes was assessed as follows: 0 = disease-free; 5 = a few, small, and isolated lesions; 15 = a few lesions affecting 10–30% of the internode surface; 40 = many lesions affecting 30–50% of the internode surface; 70 = many lesions affecting 50–75% of the internode surface; and 100 = many lesions affecting the entire internode.

Observed disease incidence on leaves (DIL) and shoots (DIS) was calculated on a 0–1 scale at each assessment date as the average proportion of internodes or leaves with PCLS symptoms, respectively, irrespective of disease severity (Madden et al., 2007). Observed disease severity was calculated by averaging the ratings of leaves (DSL) and all internodes (DSS).

### Data Analysis

A first analysis was conducted to evaluate the ability of the model to correctly predict the progress of PCLS incidence in the different epidemics. For this purpose, the model was operated by using the weather data starting from grapevine budbreak (i.e., GS = 01). Because we lacked information on the inoculum dose in the different vineyards and years, the modulator *k* in equation (1) was estimated empirically based on observed PCLS incidence at the end of the season (Table 2). In a first step, we ran the model with *k* = 1 so as to obtain a final value of S5 (*k* = 1) for leaves and shoots; in a second step, we rescaled this final value to the final PCLS incidence in the vineyard (DIL and DIS). For instance, for epidemic PI-2019 (Table 2), the sum of final disease incidence for leaves (0.370) and shoots (0.470) was 0.830 and S5(*k* = 1) = 0.742; therefore, *k* was estimated as: 0.830/0.742 = 1.10. The root mean square deviation (RMSD), the coefficient of residual mass (CRM), and the concordance correlation coefficient (CCC) were calculated (Nash and Sutcliffe, 1970; Lin, 1989; Madden et al., 2007; Piñeiro et al., 2008). For CCC calculation, the *epi.ccc* function of the “epiR” package in R v. 4.0.4 (R Core Team, 2022; Stevenson, 2022) was used. The squared sum of predictive error of a linear regression of observed PCLS incidence on leaves or shoots (DIL or DIS) versus predicted data (S5 on leaves or S5 on shoots) was decomposed by calculating Theil’s partial inequality (Theil’s U statistic) coefficients, which distinguish between different sources of predictive error: *U_bias_* is the proportion of predictive error associated with the mean differences between the observed and predicted; *U_slope_* is the proportion of predictive error associated with the deviations from the 1:1 line; and *U_error_* is the proportion of predictive error associated with the unexplained variance. The sum of these coefficients is 1 (Smith and Rose, 1995; Piñeiro et al., 2008).

A second analysis was conducted (i) to evaluate the model’s ability to predict *D. ampelina* infection events and (ii) to determine the best thresholds of model output for predicting infection events. For this purpose, the model was operated as indicated before, with *k* = 1 for any epidemic. A receiver operating characteristic (ROC) curve analysis was conducted using the *roc* function of the “pROC” package (Robin et al., 2011). For either leaves or shoots, field assessments were categorized as either 0 (no increase of PCLS severity relative to the previous assessment) or 1 (increase in PCLS severity relative to the previous assessment). Model outputs were in turn categorized as 0 or 1 depending on whether or not the accumulated values of *INFR_L_* or *INFR_S_* in an incubation period between two assessments were > 0, with the incubation periods of 12 days for leaves and 18 days for shoots. The ROC analysis measures the accuracy of different cut-off values as predictors; in this work, the cut-off values were the different values of the model outputs associated to all the field assessment performed in the 11 epidemics. ROC curves plot the specificity values (true negative proportion of cases; i.e., the cases in which no infection was predicted by the model and no disease was observed in the field) and 1-sensitivity values (true positive proportion of cases; i.e., the cases in which disease was predicted by the model and was observed in the field) associated with all of the possible cut-off thresholds of the model output (Madden, 2006; Madden et al., 2007). The best threshold is the point of the ROC curve closest to the upper left corner, i.e., the threshold with the combination of highest specificity and sensitivity. The area under the ROC curve (AUROC) and its confidence interval (CI) were also calculated; AUROC values between 0.5 and 1 indicate that the model output is a good predictor of the increase in PCLS in the field.

### Results of Model Validation

In the Italian vineyards, the spring of 2019 was mild and rainy, with an average temperature ranging from 15.1 to 17.0°C (minimum 7.7–8.5°C and maximum 26.4–33.2°C), and with >250 mm of rain accumulated from April to June. Hours of wetness accumulated in spring ranged from 426 h in PI-2019 to 759 h in SO-2019 (Supplementary Figure 1). Observed PCLS incidence was higher in PI-2019 (the proportion of leaves and shoots affected on a 0–1 scale was 0.36 and 0.47, respectively) than in SO-2019 (0.11 and 0.23), BS-2019 (0.04 and 0.11), or DO-2019 (0.08 and 0.06). Similarly, observed PCLS severity was higher in PI-2019 (2.3 and 6.4% of the leaf and shoot area with symptoms, respectively) than in SO-2019 (0.5 and 1.2%), BS-2019 (0.2 and 0.9%), or DO-2019 (0.5 and 0.4%; Table 2).

In 2020, the weather was drier in Piacenza than in the other Italian vineyards; in PI-2020, there were 172 mm of rain and only 276 h of accumulated wetness; and in the other vineyards, there were > 190 mm of rain and > 500 h of wetness. In BS-2020, there were > 800 h of wetness. Consequently, the observed PCLS incidence and severity were lower in 2020 than in 2019 in PI-2020 but were higher in 2020 than in 2019 for the other vineyards (Table 2).

In the Montenegrin vineyards, the spring of 2020 was less rainy than in the Italian vineyards, with 100–140 mm rain and 445–694 h of wetness; temperatures were mild and similar in vineyards from both countries (Supplementary Figure 2). PCLS was overall higher in the Montenegrin than in the Italian vineyards, probably due to a higher level of *D. ampelina* inoculum in the former vineyards. PCLS incidence ranged from 0.26 to 0.35 for leaves and from 0.20 to 0.36 for shoots; PCLS severity ranged from 7.0 to 3.9% for leaves and shoots, respectively, in PO-2020; from 8.4 to 1.9% in GO-2020; and from 20.5 to 7.0% in BP-2020 (Table 2).

**Figure 2:**
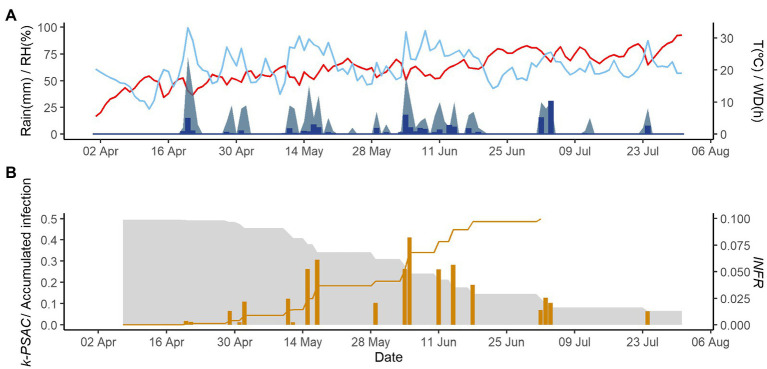
Model output for the vineyard in Piacenza in 2020. **(A)** Weather data: air temperature (T, red line in °C), rain (blue bars in millimeters), wetness duration (WD, light blue area in hours), and relative humidity (RH, blue line in %). **(B)** The gray is represent the inoculum dose as *k*-*MATR* (gray area), daily values of predicted infection (orange bars; average value of *INFR_L_* and *INFR_S_*), and accumulated infection during the season (orange line).

**Figure 3:**
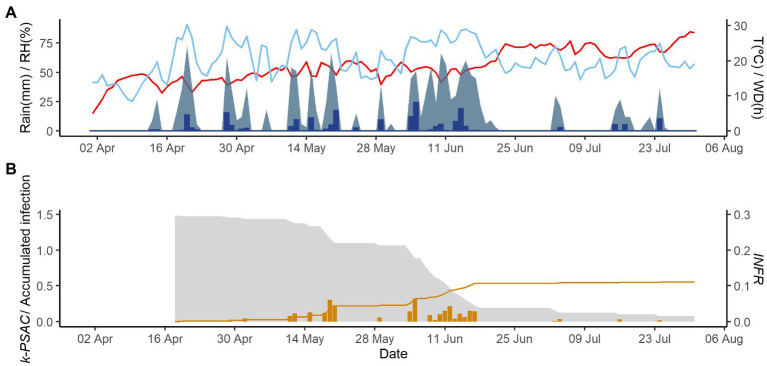
Model output of PCLS for the vineyard in Domini in 2020. **(A)** Weather variables: air temperature (T, red line in °C), rain (blue bars in millimeters), leaf wetness duration (WD, light blue area in hours), and relative humidity (RH, blue line in %). **(B)** Inoculum dose as *k*-*MATR* (gray area), daily values of predicted infection (orange bars; average value of *INFR_L_* and *INFR_S_*), and accumulated infection during the season (orange line).

**Figure 4:**
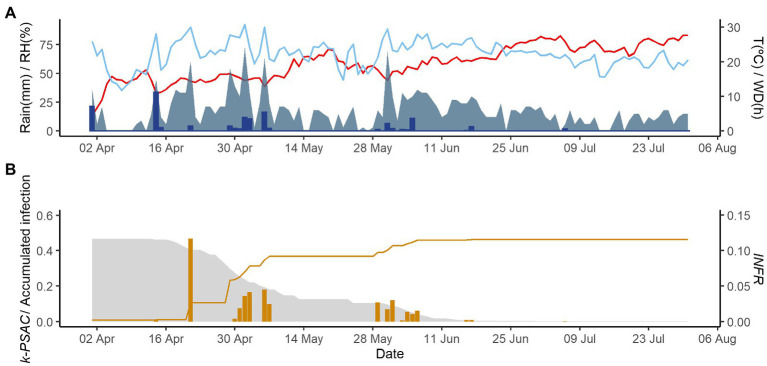
Model output of PCLS for the vineyard in Godijne in 2020. **(A)** Weather variables: air temperature (T, red line in °C), rain (blue bars in millimeters), leaf wetness duration (WD, light blue area in hours), and relative humidity (RH, blue line in %). **(B)** Inoculum dose as *k*-*MATR* (gray area), daily values of predicted infection (orange bars; average value of *INFR_L_* and *INFR_S_*), and accumulated infection during the season (orange line).

Figures 2–4 show examples of the model output for the epidemics of PI-2020, DO-2020, and GO-2020, respectively. In PI-2020, budbreak occurred in early April, and the inoculum was predicted to be present between April and the end of July (Figure 2B); in this period, 19 infection events were predicted (orange bars in Figure 2B). In DO-2020, budbreak of the vines occurred in mid-April, and most of the inoculum was dispersed before mid-June; infections were mainly predicted in mid-May and mid-June (Figure 3B). In GO-2020, budbreak of the vines occurred in early April, and most of the inoculum was dispersed between mid-April and mid-May; infections were predicted in early May and early June (Figure 4B).

**Figure 5:**
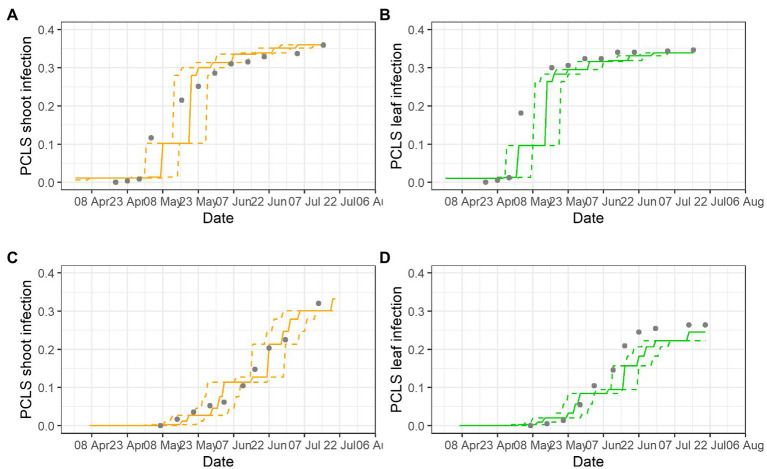
Comparison of model output and PCLS incidence observed on shoots **(A,C)** and leaves **(B,D)** in epidemics BP-2020 **(A,B)** and PI-2020 **(C,D)**. Dots represent the observed PCLS incidence; lines represent the predicted disease progress on leaves (unbroken green lines) or shoots (unbroken orange lines), and their prediction interval based on the variability of the incubation length (dotted lines).

Examples of model validation for two epidemics are provided in Figure 5; gray dots show disease assessments, lines are model outputs for disease progress (as S5 on leaves and shoots), and dotted lines show the prediction interval based on the variability in the incubation length. Most of the observed PCLS incidence was within the prediction range of the model. In BP-2020, the model correctly predicted the substantial increase of PCLS incidence in shoots with values ranging from 0.11 at the beginning of May, to 0.21 at mid-May, and to 0.31 at the beginning of June; after that, the model correctly predicted the slow increase of PCLS incidence to a value of 0.36 at the end of July (Figure 5A). For leaves, disease increase in BP-2020 in early May was underestimated, but then, the model correctly predicted the PCLS incidence from mid-May to mid-July (Figure 5B). In PI-2020, the pattern of observed PCLS incidence differed on leaves vs. shoots, and the model correctly predicted these differences. For shoots, the model predicted an increase from the beginning of June (PCLS incidence of 0.104) to mid-July (PCLS incidence of 0.407), and this pattern was consistent with observed data (Figure 5C). For leaves, the model predicted the increase of PCLS symptoms mostly on June (PCLS incidence ranging from 0.084 to 0.220), and this pattern was also consistent with observed data (Figure 5D); although the model underestimate PLCS incidence predictions from mid-June to the end of the season, this underestimation was always <0.05, with average of 0.012 (on a 0 to 1 scale).

In our comparisons between observed and predicted data for PLCS infection in all 11 epidemics, the values of CCC were 0.913 for leaves and 0.937 for shoots, and values for RMSD and CRM were always <0.09 (Table 3). As indicated by Theil’s statistic, most of the errors were associated with unexplained variance (73% for leaves and 90% for shoots), although a higher proportion of errors was attributed to bias in the case of leaves (26%) than in the case of shoots (7%). When both datasets (leaves and shoots) were merged, the CCC was 0.925, RMSD was 0.055, and CRM was 0.005. The Theil’s statistic showed that 88% of the errors corresponded to unexplained variance, indicating that the model did not have a pattern of over- or underestimating predictions (Table 3).

**Table 3:** Overall goodness-of-fit and Theil’s U statistic for the PCLS infection (of leaves, shoots, and both leaves and shoots) predicted by the model and the disease incidence observed in the vineyards of Table 2.

Organ	Goodness-of-fit^a^			Theil’s U statistic^b^		
	RMSD	CRM	CCC (CI)	Ubias	Uslope	Uerror
Leaves	0.064	−0.084	0.913 (0.872–0.941)	0.26	0.01	0.73
Shoots	0.045	0.093	0.937 (0.907–0.958)	0.07	0.04	0.90
Both	0.055	0.005	0.925 (0.901–0.943)	0.04	0.08	0.88

a RMSD: root mean square deviation; CRM: coefficient of residual mass; and CCC: concordance correlation coefficient with its confidence interval (CI) in brackets.

b Theil’s U statistic coefficients distinguish between sources of predictive error: Ubias is the proportion of error associated with mean differences between the observed and predicted; Uslope is the proportion of error associated with the deviations from the 1:1 line; and Uerror is the proportion of error associated with unexplained variance.

Regarding the ROC analysis, the use of the model for predicting *D. ampelina* infection events resulted in an AUROC value of 0.716 (CI 0.6110–0.822) for leaves and 0.749 (CI 0.655–0.843) for shoots, in both cases with CI values >0.5. For both shoots and leaves, the best cutting point of the curve (the part of the curve that is closest to the upper left corner, i.e., the point with the highest specificity and sensitivity) was at INFR_L_ or INFR_S_ = 0.013, which can then be considered the best predictor of *D. ampelina* infection.

## Discussion

In this study, we developed a mechanistic model of *D. ampelina* infection by using previously reported data and knowledge obtained by *in planta* experiments conducted under controlled conditions (Erincik et al., 2001, 2003) and by field experiments conducted in multiple years and locations (Anco et al., 2012, 2013; González-Domínguez et al., 2021). The model is dynamic, because its predictions are driven by weather and other external variables (Teng et al., 1980; Leffelaar, 1993; Rossi et al., 2010).

Our model can also be considered to be pathogen-focused, because it uses systems analysis (Leffelaar, 1993; Rossi et al., 2010) to describe and simulate the main stages of the *D. ampelina* life cycle that lead to disease onset and progress during the grape-growing season. These stages include (i) the overwintering and maturation of pycnidia on affected canes and rachises, (ii) the dispersal of alpha conidia to leaves and shoots, (iii) the infection, and (iv) the onset of disease symptoms.

The model was validated with an independent dataset (i.e., data not used in model development) of 11 PCLS epidemics. In this validation, we compared the observed versus the predicted PCLS incidence; the high CCC values (>0.91) and the low weight of errors (RMSD<0.06 and CRM < 0.09) indicate that the model correctly predicts PCLS development in vineyards. The decomposition of the errors indicated that most errors could be attributed to unexplained variance rather than to model bias (Smith and Rose, 1995; Piñeiro et al., 2008). Because the dataset used for validation included multiple locations and years, with a wide range of climatic conditions, grapevine cultivars, agronomical management, and disease incidence and severity, the model may be considered accurate (i.e., it provides predictions close to reality) and robust (i.e., it provides accurate predictions under a range of environmental, agronomical, and epidemiological conditions; Rossi et al., 2010). The ROC curve analysis confirmed the ability of the model to predict the increase of PCLS severity in the field, indicating that it could be used by growers for risk-based decision making for disease control (Zadoks and Rabbinge, 1989; Madden, 2006).

Although the model was mainly developed based on well-documented data and a well-documented understanding of the disease, we needed to make assumptions because of knowledge gaps regarding the quantification of primary inoculum and the effect of the environment on the length of the incubation period.

For the primary inoculum, the model modulates the primary inoculum dose in a vineyard through the *k* parameter of equation (1). This parameter depends on several difficult-to-estimate factors, including the incidence and severity of PCLS on shoots in the previous season, the proportion of affected shoots remaining on the trellis after pruning, and the effect of the environment on the production of new pycnidia during fall and winter (Anco et al., 2013; Wilcox et al., 2015; González-Domínguez et al., 2021). For a precise parametrization of *k*, the effect of disease, environment, and agronomical practices on the dose of inoculum inherited from the previous season should be studied and incorporated in the model (Leffelaar, 1993). Alternatively, methods should be developed for a precise quantification of the potential inoculum, as has been done for ascospores of *Venturia inaequalis* in apple leaf residue (parameter “PAD” on MacHardy and Jeger, 1983) or for oospores of *Plasmopara viticola* in grape leaf residue (Si Ammour et al., 2019). To validate the model’s ability to predict disease progress in the current research, we estimated *k* empirically in the different vineyards and years, with values ranging 0.45–2.25 (Table 2). The magnitude of *k* should affect the magnitude of model predictions, but not the shape of the predicted disease progress; therefore, the model validation in terms of predicted versus observed PCLS incidence is valid regardless the magnitude of *k*. In addition, the model’s ability to correctly predict infection periods was validated by setting *k* = 1 in a ROC analysis, as has been previously done for other models (Rossi et al., 2008; Ji et al., 2021). Therefore, in the absence of more precise information about the inoculum dose in a vineyard, the model can be used to predict PCLS infection events during the season by using *k* = 1.

For the length of the incubation period, the model assumes a number of days from infection to the appearance of symptoms on leaves or shoots (Erincik et al., 2002; Nita et al., 2006b). It is well known that *D. ampelina* growth and incubation length are influenced by environmental conditions (Wilcox et al., 2015); for instance, the growth of the fungus on potato dextrose agar is influenced by temperature, with cardinal temperatures of 5–35°C and an optimum of 20°C (González-Domínguez et al., 2021). In a preliminary analysis, however, we found that the number of days provided a better estimate of the incubation length in vineyards than temperature-dependent equations (see Supplementary Material). For a better estimate of the temperature-dependent length of incubation, studies should be conducted with artificial inoculation of *D. ampelina* alpha conidia *in planta* and with subsequent incubation at different temperatures. For decision making for the control of a monocyclic disease like PCLS, however, a precise estimation of the incubation length may be much less important than the correct prediction of infection; this is because the onset of a monocyclic disease is not related to the production of secondary inoculum that can generate new infections during the season.

The model was validated in vineyards with no fungicide treatments against PCLS; fungicides were, however, used to control other diseases; and for example, azoxystrobin, cymoxanil, folpet, metiram, or zimoxanile were used to control downy mildew in some cases. These treatments should have had only minor effects or no effect on our model validation for three reasons: (i) these fungicides are not able to completely control *D. ampelina* (Nita et al., 2006b; Wilcox et al., 2015); (ii) the possible effects of these fungicides were to some extent accounted for in the empirical estimation of the *k* parameter; and (iii) our prediction of infection events was qualitative (infected or not infected) rather than quantitative (i.e., we did not predict the severity of disease severity resulting from an infection).

In conclusion, by using the threshold of the model output that minimizes the proportion of false positives/false negatives (Madden, 2006), the model presented here may correctly predict *D. ampelina* infection events in vineyards. It follows that the model has the potential to guide the scheduling of fungicide applications for the control of PCLS in vineyards (Rossi et al., 2019). The practical use of the model should be confirmed by specific experiments that compare risk-based (model-based) application of fungicides with the usual calendar-based application of fungicides (Rossi et al., 2019, 2021). Experiments should also be conducted to find new ways to control PCLS; the ban of mancozeb in the EU and the increasing worldwide restrictions on the use of the broad-spectrum fungicides that are commonly used against PCLS represent new challenges in the management of the disease (Topping et al., 2020; Duarte et al., 2021; Rossi et al., 2021). QoI and DMI fungicides have been tested in only a few experiments, with inconsistent results, and almost no information exists about biological control agents (Wilcox et al., 2015).

Once it practically is evaluated, the model could be easily integrated into vite.net,^1^ a decision support system (DSS) that already includes models of downy and powdery mildews, Botrytis bunch rot, and black rot (González-Domínguez et al., 2020). The integration of the model into this DSS could greatly improve IPM in the vineyard by helping growers determine when a fungicide application or other treatment is needed based on the processes (predicted by epidemiological models) that lead to the development of multiple diseases (Rossi et al., 2019).

## Data Availability Statement

The raw data supporting the conclusions of this article will be made available by the authors, without undue reservation.

## Author Contributions

EG-D, TC, and VR contributed to conception and design of the model. AP, LM, LL, NL, and JL obtained the field and weather data and organized the database for model validation. EG-D and VR wrote the manuscript. All authors contributed to the article and approved the submitted version.

## Funding

The Project “Share web-based Decision Support Systems to increase the sustainability of the viticultural sector in Montenegro and Italy (VITISUST)” started in August 2018 and is supported by Foreign Affairs Ministry (IT) and Ministry of Science (ME) within the International collaboration framework between Italy and Montenegro.

## Conflict of Interest

EG-D was employed by Horta s.r.l., a spin-off company of the Università Cattolica del Sacro Cuore (Piacenza, Italy).

The remaining authors declare that the research was conducted in the absence of any commercial or financial relationships that could be construed as a potential conflict of interest.

## Publisher’s Note

All claims expressed in this article are solely those of the authors and do not necessarily represent those of their affiliated organizations, or those of the publisher, the editors and the reviewers. Any product that may be evaluated in this article, or claim that may be made by its manufacturer, is not guaranteed or endorsed by the publisher.

## Supplementary Material

The Supplementary Material for this article can be found online at: https://www.frontiersin.org/articles/10.3389/fpls.2022.872333/full#supplementary-material

## Footnotes

^1^ https://www.horta-srl.it/en/servizi/dss/
