# Supplementary material for: Development and Validation of a Mechanistic Model That Predicts Infection by Diaporthe ampelina, the Causal Agent of Phomopsis Cane and Leaf Spot of Grapevines
Source: Front Plant Sci. 2022 Apr 7;13:872333. doi: 10.3389/fpls.2022.872333 (PMC9021785; doi:10.3389/fpls.2022.872333)
Supplement: Supplementary file 1 [file Data_Sheet_1.docx]

Supplementary Material

- 1. **Calculation of the shoot-to-leaf-area ratio**

The model assumes that the alpha conidia of *Diaporthe ampelina* dispersed by splashing rain deposit on shoot and leaf surfaces, at a deposition rate (DEPR; Fig. 1 of the main manuscript), which depends on the shoot and leaf area (SA and LA, respectively) at the time of dispersal, and on a shoot-to-leaf-area ratio. SA and LA (both in cm^2^) depends on the time (in days) after budbreak (DAB).

Into the model, the shoot area is calculated as $\mathrm{SA}=r \pi$ SL, in which *r* is the average radius of the shoot (in cm), and SL is the shoot length (SL, in cm), both depending on DAB as in Eltom et al. (2013).

The relationship between *r* and DAB is calculated as:

$$r=\sqrt{\frac{0.00419 DAB+0.496}{\pi}}$$

The relationship between SL and DAB is calculated as:

$\mathrm{SL}=\frac{\mathrm{SLmax}}{1+e^{5.0-0.057 \mathrm{DAB}}}$

in which SLmax is the maximum length of the shoot (i.e., the length of the shoot at 180 DAB).

The leaf area is calculated as a function of the shoot length (SL) by using the data from Costanza et al. (2004), as follows:

when SL<12 cm; $LA=1.068 SL-0.57$

when SL≥12 cm; $\mathrm{LA}=17.838 \mathrm{SL}-245.95$

- 1. **Calculation of infection rate**

The rate of *Diaporthe ampelina* infection was calculated with data from *in planta* experiments conducted by Einrik et al. (2003). Briefly, the authors inoculated shoots and leaves of ‘Catawba’ (*Vitis labrusca*) and ‘Seyval’ (French hybrid) grapes with conidia of *D. ampelina*, and incubated them at different temperature (T; 5, 10, 15, 20, 25, 30, and 35°C) × wetness duration (WD; 5, 10, 15, and 20 h) combinations. Disease severity on leaves was assessed by estimating the number of lesions and disease severity on internodes was assessed by estimating the proportion of the area covered by lesions. Disease severity data were rescaled in a 0 to 1 scale (by dividing each value by the maximal severity observed in the experiment) and averages of the 2 varieties were calculated.

These data were used as the response variable for each temperature × wetness duration combination. Different equations were fit to these data, and the one to be used into the model was determined following the AIC criterion (Burnham and Anderson, 2002).

The combined effect of temperature and wetness duration on leaf infection was described by a β-Gompertz equation (Madden et al., 2007) in the following form:

*y* = *a* Teq*^b^* (1 – Teq)*^c^* exp(*d* exp(*e* WD))

in which: *y* is the rate of leaf infection (*INFR_L_* in the main text); *a*, *b*, *c*, *d*, and *e* are the equation parameters; WD is the wetness duration; and Teq is an equivalent of temperature calculated as Teq = (T – Tmin)/(Tmax – Tmin), in which T is the temperature regime, and Tmin and Tmax are minimal and maximal temperatures (5 and 35°C, respectively). Parameter estimates were as follows: *a* = 11.228, *b* = 1.553, *c* = 1.842, *d* = -9.336, and *e* = -0.258, with *R^2^* =0.965 and *CCC* = 0.982 (confidence interval 0.9363-0.991) (Figure 1).

The combined effect of temperature and wetness duration on shoot infection was described by a Weibull-lineal equation (Duthie, 1997) in the following form:

*y* = (((*h* + 1)/*h*) *h* ^(1/(^*^h^* ^+ 1))^ × ((exp(((T - 15) × *g*)/(*h* + 1)))/(1+exp((T-15) *g*)))) WD/WDmax

in which: *y* is the rate of shoot infection (*INFR_S_* in the main text); *h* and *g* are the equation parameters; T is the temperature regime; WD is the wetness duration and WDmax is 20 hours. Parameter estimates were as follows: *h* = 0.441 and *g* = 0.591, with *R^2^* = 0.965 and *CCC* = 0.936 (confidence interval 0.869-0.970) (Figure 2).

All equation parameters were estimated by using the *nls* function of the R ‘stats’; the *epi.ccc* function of the R ‘epiR’ package (Stevenson et al., 2012) was used to calculate the concordance correlation coefficient (CCC) (Lin, 1989).


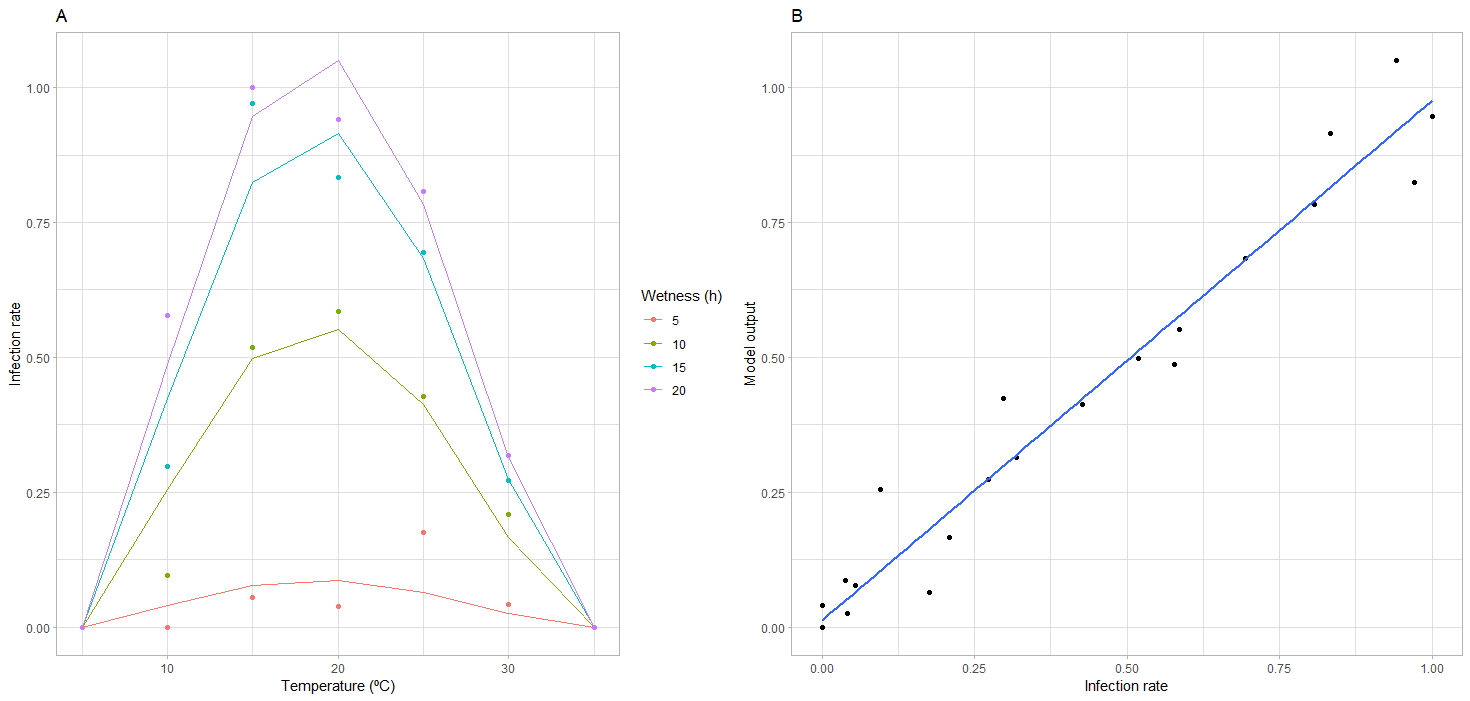


**Supplementary Figure 1.** Relationship between temperature, wetness duration and infection rate of *Diaporthe ampelina* on grapevine leaves. **A**, dots represent observed data and lines are the predicted value based on the β-Gompertz equation; **B**, plot of predicted versus observed data.


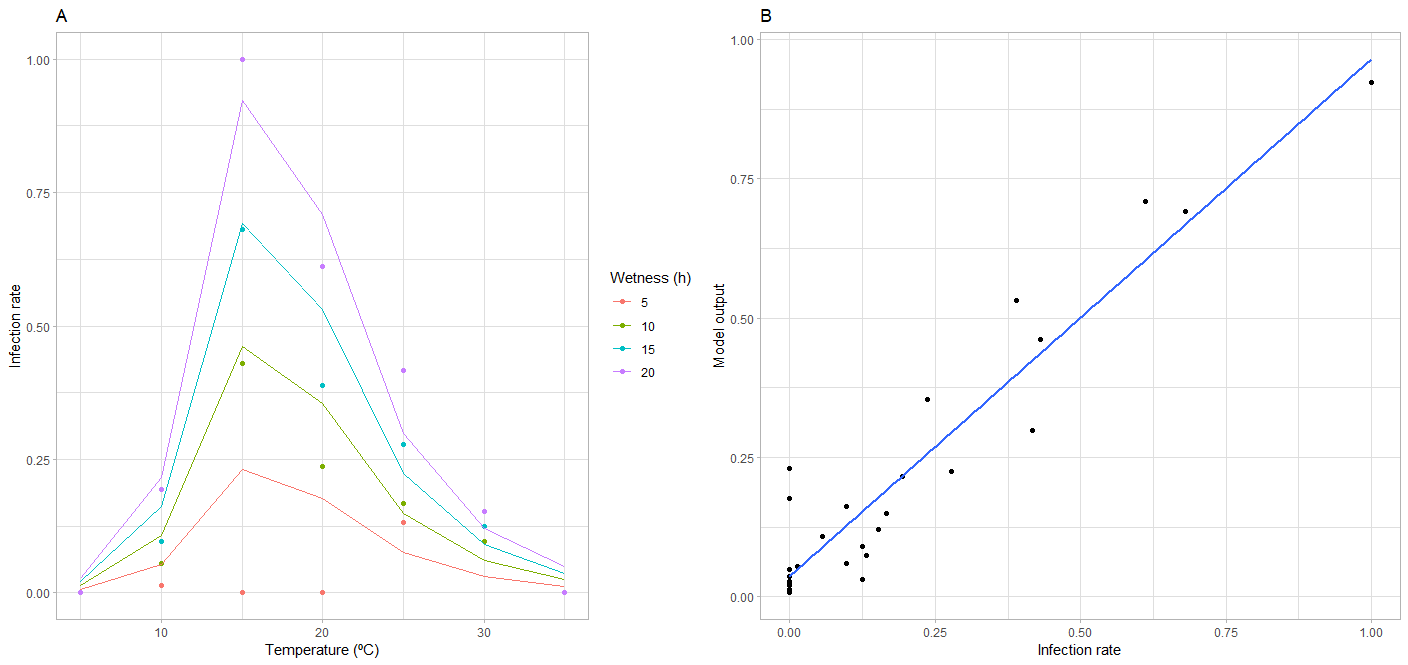


**Supplementary Figure 2.** Relationship between temperature, wetness duration and infection rate of *Diaporthe ampelina* on grapevine shoots. **A**, dots represent observed data and lines are the predicted value based on the Weibull-lineal equation developed; **B**, plot of predicted versus observed data.

**1.3. Calculation of incubation length**

Into the model, the incubation period is the time elapsing between infection and the onset of symptoms on leaves or shoots. The effect of environment and host growth stage on the length of incubation period is not well understood, and specific experiments under controlled conditions have never been conducted (Erincik et al. 2001; Erincik et al. 2003).

For the calculation of the length of the incubation period we used the disease severity assessments for the 11 epidemics in Table 1 of the main text. For each infection event predicted by the model (i.e., a day or consecutive days with S4>0.0009) we accumulated the number of days (DAYS) or the thermal time (TT) until the time when an increase of the disease severity was observed, by considering a minimum of 3 and 7 days for leaves and shoots, respectively (Erincik et al., 2002; Nita et al., 2006). An example is shown in Figure 3.

The thermal time (TT) consists on the accumulation of temperature-dependent rate of mycelial growth of *D. ampelina*, as estimated by González-Domínguez et al. (2021) through the following equation: TT_i_ = *79.93* Teq_i_*^3.889^* (1–Teq_i_)^2.83^, in which Teq_i_ is an equivalent of temperature for each i^th^ day after predicted infection, calculated as Teq_i_= (T_i_ – Tmin)/(Tmax – Tmin), where T_i_ the average temperature of the i^th^ day, and Tmin and Tmax are the minimal and maximal temperatures for mycelial growth, i.e., 5 and 35°C, respectively.


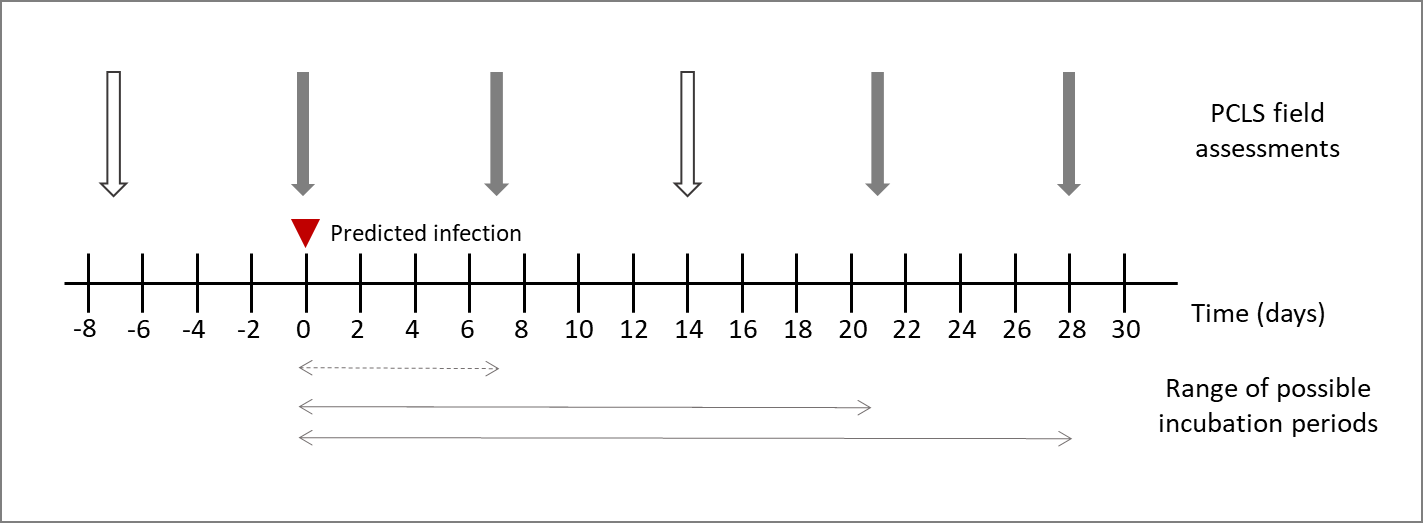


**Supplementary Figure 3.** Schematic representation of the range of possible incubation periods associated to an infection event predicted by the model. The solid black line represents the time (in days) from the predicted infection (red triangle). The vertical arrows are the moments in which field assessments were performed; grey arrows indicated an increase of PCLS incidence whereas empty arrows indicate no increase of PCLS incidence from the previous assessment. The horizontal arrows are the range of possible incubation periods; the dashed arrow indicate the minimum incubation period that can be considered.

The distribution of DAYS and TT for the 74 infection events predicted for the 11 epidemics is shown in Figure 4 for leaves and shoots. Considering the 25^th^ and 75^th^ percentiles of these distributions, the average incubation length was 18 (range of 11 to 25) and 12 (7 to 18) DAYS for shoots and leaves, respectively, or 11 (7 to 16) and 8 (3 to 12) TT, respectively.


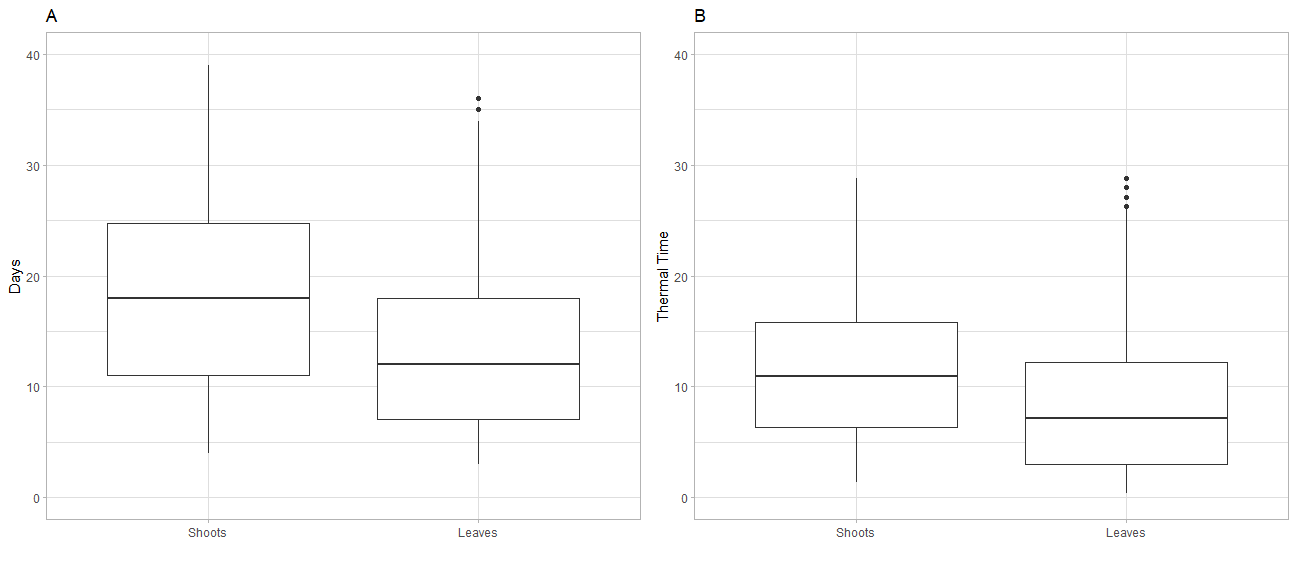


**Supplementary Figure 4.** Length of incubation in days (A) or thermal time (B) for shoots and leaves for the infection events estimated by the model in the 11 epidemics of Table 1 in the main text. The boxes show the 1st quartile, the median, and the 3rd quartile; dots are outliers.

By using the above averages for estimating the incubation length for the infection periods in the 11 epidemics, the concordance correlation coefficient (CCC) with the real data were 0.890 (CI 0.842-0.925) and 0.937 (CI 0.906-0.957) for leaves and shoots, respectively, using DAYS as predictor, which were slightly higher than 0.887 (CI 0.834-0.923) and 0.918 (CI 0.880-0.944) for leaves and shoots, respectively, using TT as predictor. Therefore, the incubation length into the model is calculated as number of days, specifically 18 days with a window of 11-25 days for shoots, and 12 days, with a windows of 7-18 days for leaves.

**References:**

Burnham, K. P., and Anderson, D. 2002. Model Selection and Multimodel Inference: A Practical Information-Theoretic Approach, 2nd ed. Springer,New York

Costanza, P., Tisseyre, B., Hunter, J.J., and Deloire, A. 2004. Shoot development and non-destructive determination of grapevine (Vitis vinifera L.) leaf area. S. Afr. J. Enol. Vitic. 25: 43-47.

Duthie, J. A. 1997. Models of the response of foliar parasites to the combined effects of temperature and duration of wetness. Phytopathology 87: 1088–1095.

Eltom, M., Trhought, M., and Winefield, C.2013. The effects of cane girdling before budbreak on shoot growth, leaf area and carbohydrate content of Vitis vinifera L. Sauvignon Blanc grapevines. Fun. Plant Biol. 40: 749–757.

Erincik, O., Madden, L.V., Ferree, D.C., Ellis, M.A. 2001. Effect of growth stage on susceptibility of grape berry and rachis tissues to infection by *Phomopsis viticola*. Plant Dis. 85: 517–520.

Erincik, O., Madden, L.V., Ferree, D.C., and Ellis, M.A. 2002. Infection of grape berry and rachis tissues by *Phomopsis viticola*. Plant Heal. Prog. 3: 8.

Erincik, O., Madden, L.V., Ferree, D.C., and Ellis, M.A. 2003. Temperature and wetness-duration requirements for grape leaf and cane infection by *Phomopsis viticola*. Plant Dis. 87: 832–840.

González-Domínguez, E., Caffi, T., Languasco, L., Latinovic, N., Latinovic, J., and Rossi, V. 2021. Dynamics of *Diaporthe ampelina* conidia released from grape canes that overwintered in the vineyard. Plant Disease.

Lin, L. 1989. A concordance correlation coefficient to evaluate reproducibility. Biometrics 45: 255–268.

Madden, L.V., Hughes, G., and Van den Bosch, F. 2007. The Study of Plant Disease Epidemics. American Phytopathological Society, St. Paul, MN, U.S.A.

Nita, M., Ellis, M.A., Wilson, L.L. and Madden, L.V. 2006. Evaluation of a disease warning system for Phomopsis cane and leaf spot of grape: a field study. Plant Dis. 90: 1239–1246.

Stevenson, M. 2012. Epir, An R package for the analysis of epidemiological data. R package version 09-43.

## 2.1. Supplementary Figures

**Supplementary Figure 5.** Weather conditions from April to July of 2019 and 2020 in the four Italian vineyards used for model validation. The figure shows daily values of temperature (T, red line, in ºC), wetness duration (WD, light blue area, in hours), and rain (blue bars, in millimeters). Location and main characteristics of the vineyards are in Table 2 of the main document.

**Supplementary Figure 6.** Weather conditions from April to July of 2020 in the three Montenegrin vineyards used for model validation. The figure shows daily values of temperature (T, red line, in ºC), wetness duration (WD, light blue area, in hours), and rain (blue bars, in millimeters). Location and main characteristics of the vineyards are in Table 2 of the main document.
