# Supplementary figures and images for: Development and Validation of a Mechanistic Model That Predicts Infection by Diaporthe ampelina, the Causal Agent of Phomopsis Cane and Leaf Spot of Grapevines
Source: Front Plant Sci. 2022 Apr 7;13:872333. doi: 10.3389/fpls.2022.872333 (PMC9021785; doi:10.3389/fpls.2022.872333)

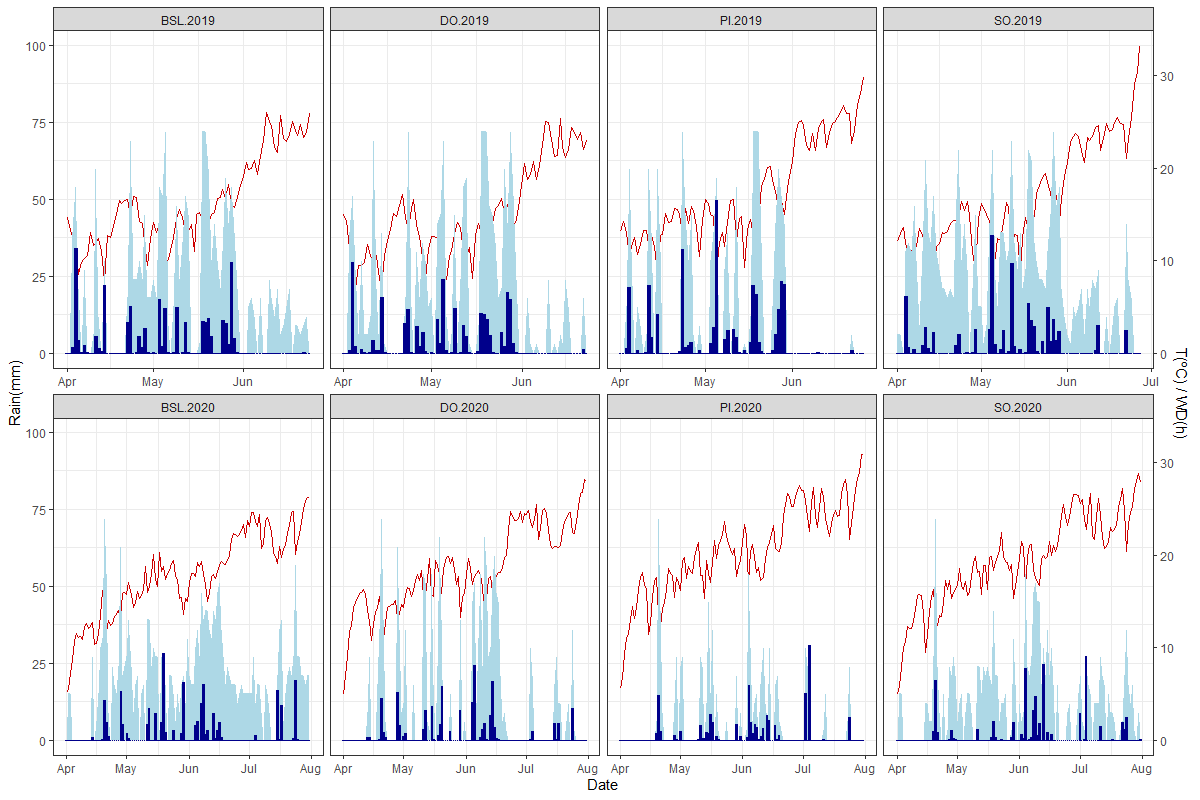

Supplement: Supplementary file 2 [file Image_1.TIFF]

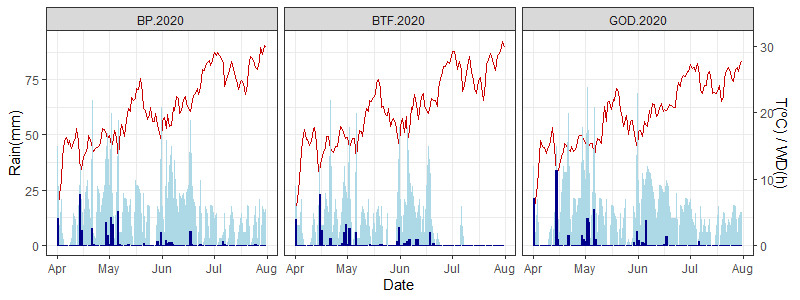

Supplement: Supplementary file 3 [file Image_2.TIFF]
